# Matrix-Assisted Laser Desorption Ionization-Time of Flight Mass Spectrometry for the Identification of Clinically Relevant Bacteria

**DOI:** 10.1371/journal.pone.0016424

**Published:** 2011-01-25

**Authors:** Cinzia Benagli, Viviana Rossi, Marisa Dolina, Mauro Tonolla, Orlando Petrini

**Affiliations:** 1 Cantonal Institute of Microbiology, Bellinzona, Switzerland; 2 Microbiology Unit, Plant Biology Department, University of Geneva, Switzerland; University of Hyderabad, India

## Abstract

**Background:**

Matrix-assisted laser desorption ionization-time of flight mass spectrometry (MALDI-TOF MS) allows rapid and reliable identification of microorganisms, particularly clinically important pathogens.

**Methodology/Principal Findings:**

We compared the identification efficiency of MALDI-TOF MS with that of Phoenix®, API® and 16S ribosomal DNA sequence analysis on 1,019 strains obtained from routine diagnostics. Further, we determined the agreement of MALDI-TOF MS identifications as compared to 16S gene sequencing for additional 545 strains belonging to species of *Enterococcus*, *Gardnerella*, *Staphylococcus*, and *Streptococcus*. For 94.7% of the isolates MALDI-TOF MS results were identical with those obtained with conventional systems. 16S sequencing confirmed MALDI-TOF MS identification in 63% of the discordant results. Agreement of identification of *Gardnerella*, *Enterococcus*, *Streptococcus* and *Staphylococcus* species between MALDI-TOF MS and traditional method was high (Crohn's kappa values: 0.9 to 0.93).

**Conclusions/Significance:**

MALDI-TOF MS represents a rapid, reliable and cost-effective identification technique for clinically relevant bacteria.

## Introduction

Matrix-assisted laser desorption ionization-time of flight mass spectrometry (MALDI-TOF MS) is rapidly attracting the interest of microbiologists working in the routine labs, because of its powerful features that allow rapid and reliable identification of microorganisms.

Standardized test systems such as API® and VITEK® 2 (bioMérieux), or PHOENIX® (BD Diagnostics), complemented by traditional culture and microscopy methods, have so far been used in routine labs for the rapid identification of clinical microorganisms. With the introduction of these methods, the average time needed for a reliable and validated identification ranged from 6 h to 18 h and in the last few years, sequence analysis of small-subunit rRNAs or selected genes by PCR methods has complemented the biochemical methods, additionally decreasing throughput time and becoming in several cases the gold standard [1].

The recent developments of MALDI-TOF MS are rapidly changing the routine diagnostics scene. MALDI-TOF MS is a powerful method to detect and identify proteins by molecular weight determination of individual, specific fragments [2]. The method is accurate and easy to use, allowing quick determination of molecular weights of proteins with minimal sample requirements.

MALDI-TOF MS is now widely used for the identification and characterization of clinically important microorganisms [3]. The currently available identification databases target the identification of human pathogens [4] and MALDI-TOF MS represents a valid and rapid alternative to conventional methods of identification and classification of human pathogens in microbiology.

Traditionally, validation of a new identification system to be introduced in routine diagnostics consists of running parallel identifications of a large number of isolates using the new method concomitantly with set standards.

In this study we compared the identification efficiency of MALDI-TOF MS with that of Phoenix®, API® and 16S ribosomal DNA sequence analysis. In a first step we analyzed 1,019 strains obtained sequentially during three months from our routine diagnostic laboratory. In a second step, we studied in more detail 545 isolates of species belonging to the genera *Enterococcus*, *Gardnerella*, *Staphylococcus*, and *Streptococcus* and determined the agreement (and, when possible, efficiency, sensitivity and specificity) of the MALDI-TOF MS identifications as compared to 16S gene sequencing as the gold standard.

## Results

**Figure 1:**
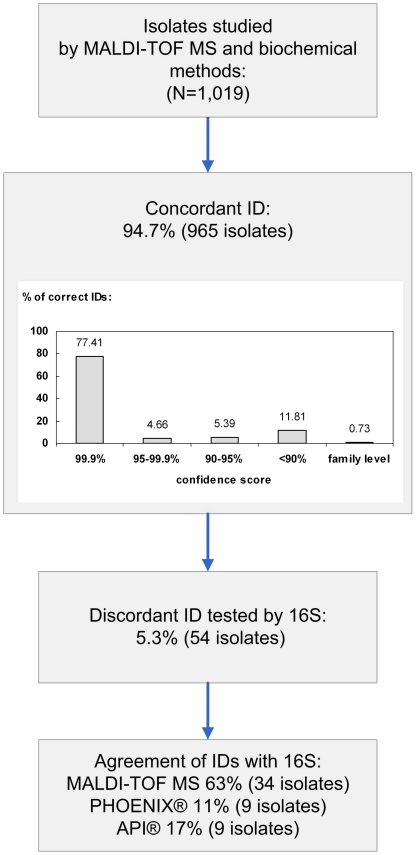
Results of the validation analysis with all isolates.

In a first step we analysed 1,019 strains obtained from the routine diagnostic lab. The results are described in Fig. 1. For 965 isolates (94.7%) the results of MALDI-TOF MS were identical with those obtained with the BD PHOENIX system and the confidence level of the MALDI-TOF MS identification was almost 100% for approximately 75% of the isolates tested. API or 16S sequencing confirmed MALDI-TOF MS identification in 63% of the discordant results. Overall, therefore, MALDI-TOF was able to identify correctly more than 98% of the isolates tested. Table 2 reports the specificity, sensitivity, PPV, NPV and efficiency values of the MALDI-TOF MS identification, compared to the classical methods, for those bacterial species for which we could analyse at least 15 isolates. With the exception of *Enterobacter cloacae* and *Klebsiella oxytoca*, for which the sensitivity values for identifications at the confidence level of at least 90% are relatively low, MALDI-TOF MS has a quite high efficiency of identification. In most cases these values are still even if the limit of acceptance of the confidence level for correct identification by MALDI-TOF MS is set at 99.9% (Table 2).

**Table 1:** Genera of bacteria studied in the first validation step.

Genus	No. of isolates
***Acinetobacter***	22
***Citrobacter***	19
***Enterobacter***	59
***Enterococcus***	39
***Escherichia***	293
***Klebsiella***	76
***Morganella***	15
***Proteus***	85
***Pseudomonas***	125
***Serratia***	23
***Staphylococcus***	182
***Stenotrophomonas***	33
***Streptococcus***	21
**Others** *	27
**Total isolates**	1019

* includes *Aerococcus* (1), *Aeromonas* (3), *Alcaligenes* (2), *Bacillus* (1), *Chryseobacterium* (2), *Corynebacterium* (3), *Delftia* (1), *Hafnia* (3), *Micrococcus* (2), *Pasteurella* (3), *Providencia* (2), *Raoultella* (1), *Shewanella* (1), *Vibrio* (1).

**Table 2:** Sensitivity, specificity, positive predictive values (PPV), negative predictive values (NPV) and efficiency of the MALDI-TOF MS identification of selected species as compared to API® and PHOENIX®.

	N	Sensitivity	Specificity	PPV	NPV	Sensitivity	Specificity	PPV	NPV	Efficiency
		(>90)	(>90)			(99.9)	(99.9)			
***Acinetobacter baumanii***	16	87.5	100	100	99.8	81.25	100	100	99.7	93.7
***Enterobacter aerogenes***	16	93.75	100	100	99.9	93.75	100	100	99.9	100
***Enterobacter cloacae***	36	69.44	100	100	98.9	30.56	100	100	97.5	97.2
***Escherichia coli***	294	95.58	100	100	98.2	90.82	100	100	96.4	98.6
***Klebsiella oxytoca***	24	79.17	100	100	99.5	62.5	100	100	99.1	91.6
***Klebsiella pneumoniae***	53	90.57	100	100	99.5	58.49	100	100	97.8	100
***Morganella morganii***	15	93.33	100	100	99.9	93.33	100	100	99.9	100
***Proteus mirabilis***	75	98.67	100	100	99.9	96	100	100	99.7	100
***Pseudomonas aeruginosa***	120	99.17	100	100	99.9	97.5	100	100	99.7	99.1
***Serratia marcescens***	23	95.65	100	100	99.9	86.96	100	100	99.7	95.6
***Stenotrophomonas maltophilia***	33	96.97	100	100	99.9	93.94	100	100	99.8	100

In parentheses: confidence level of identification. For details see text.

We analysed separately 76 isolates of *Gardnerella*, 50 of *Enterococcus*, and 76 of *Streptococcus* (mainly *S. agalactiae* and *S. pneumoniae*) by MALDI-TOF MS, API/PHOENIX and 16S sequencing. The results are described in Table 3.

**Table 3:** AI between MALDI-TOF MS and conventional methods for isolates of *Gardnerella* spp., *Enterococcus* spp. and *Streptococcus* spp.

Taxon	N	Identical ID MALDI-TOF MS – conventional methods	%AI
***Gardnerella*** ** spp.**	**76**	**74**	**97.4**
***Enterococcus*** ** spp.** * ***E. casselliflavus*** ***E. faecalis*** ***E. faecium*** ***E. gallinarum***	**50**121199	**44**021194	**88**010010044.4
***Streptococcus*** ** spp.** * ***S. agalactiae*** ***S. pneumoniae*** **Others**	**76**183028	**60**162420	**78.9**88.98071.4

For *Streptococcus* and *Enterococcus*, 16S sequencing was used as the constructed gold standard.

*16S sequencing ID.

For *Gardnerella* spp., the %AI was almost 100%. Very good concordance between 16S sequencing and MALDI-TOF MS was also observed for *Enterococcus faecalis* and *E. faecium*, for which the concordance was 100%. Results for *E. gallinarum* were not particularly good (44.4%), but the number of isolates investigated (9) was low. MALDI-TOF MS showed a fair performance also with *Streptococcus* spp., with quite high sensitivity and specificity values for *S. agalactiae* and *S. pneumoniae* (Table 4).

**Table 4:** Sensitivity, specificity, PPV and NPV for *Streptococcus agalactiae* and *S. pneumoniae*.

	Sensitivity	Specificity	PPV	NPV
***S. agalactiae***	100	96.7	88.9	100
***S. pneumoniae***	89	87.75	80	93.48

The study sample on which the calculation is based was represented by *Streptococcus* spp. only.

In a separate experiment we evaluated the efficiency of MALDI-TOF MS in the identification of *Staphylococcus* spp. We considered a sample of 343 staphylococci belonging to 17 species (*S. aureus*, *S. auricularis*, *S. capitis*, *S. carnosus*, *S. cohnii*, *S. epidermidis*, *S. equorum*, *S. haemolyticus*, *S. hominis*, *S. lugdunensis*, *S. pasteuri*, *S. saprophyticus*, *S. schleiferi*, *S. sciuri*, *S. simulans*, *S. warneri*, *S. xylosus)* and we computed sensitivity, specificity, PPV and NPV values for the four most commonly isolated taxa in our routine laboratory (*S. aureus*, *S. epidermidis*, *S. hominis*, and *S. haemolyticus*) as well as the overall %AI for all species. The outcome of the identification by MALDI-TOF MS was compared to that obtained by 16S sequencing, which for the purpose of this work was considered the gold standard, and other methods (API®, PHOENIX®) currently used in our laboratory.

**Figure 2:**
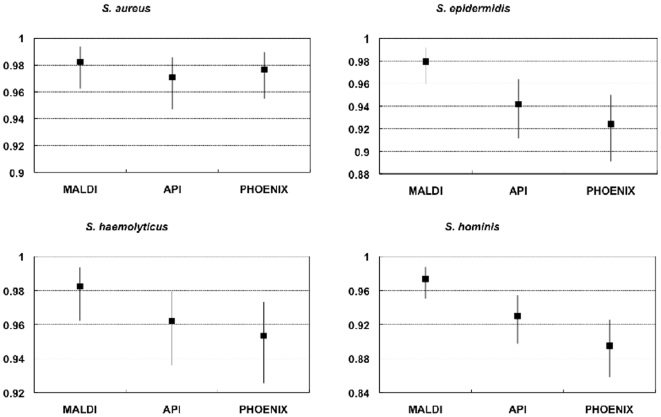
Efficiency of different methods for the identification of four *Staphylococcus* species as compared to 16S sequencing.

**Figure 3:**
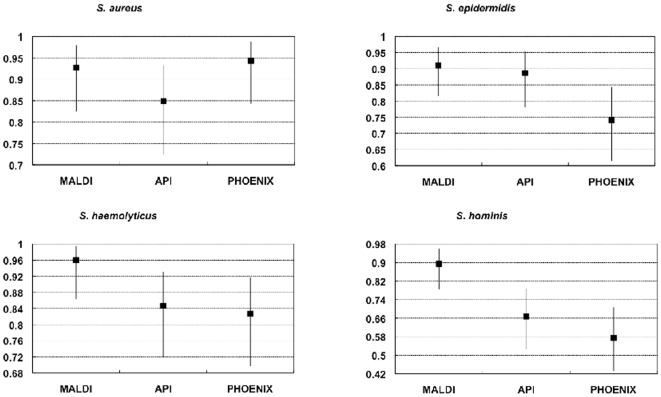
Sensitivity of different methods for the identification of four *Staphylococcus* species as compared to 16S sequencing.

**Figure 4:**
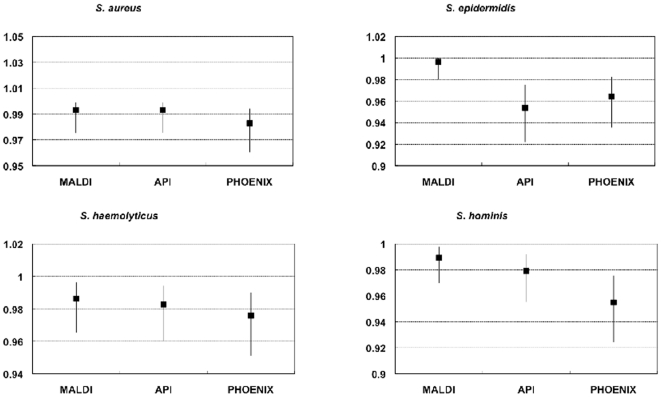
Specificity of different methods for the identification of four *Staphylococcus* species as compared to 16S sequencing.

Results are presented in Figure 2, Figure 3 and Figure 4. For the four species considered, MALDI-TOF MS was at least as good as the other methods in identifying the species studied, and mostly the sensitivity, specificity, PPV and NPV values were trend-wise, albeit not statistically significantly superior to those obtained for the other methods. The identification agreement between MALDI-TOF MS and the gold standard used, as represented by the Crohn's kappa values, ranged between 0.9 and 0.93, indicating an almost perfect agreement. The identification efficiency was also always over 90%. The %AI over the whole sample investigated was 88.5%.

## Discussion

In this study, MALDI-TOF MS has proven to be a fast, accurate and reliable technique for the identification of clinically relevant bacteria. We have observed an almost perfect agreement between identifications obtained by MALDI-TOF MS and those provided by conventional, biochemical methods. When discordant results among mass spectrometry and biochemical methods were observed, sequencing most often confirmed MALDI-TOF MS identification.

Identification of *Gardnerella* species by MALDI-TOF MS has proven to be reliable, needing no additional confirmations by other methods. The same applied to *E. faecium* and *E. faecalis*, both regularly identified by MALDI-TOF MS at confidence levels of almost 100% and with a %AI with 16S sequencing of 100%. We were not able to study enough samples of *E. caselliflavus* and *E. gallinarum* to report reliable values for MALDI-TOF MS identification of these two species; nevertheless, our present daily experience places MALDI-TOF MS reliability at least at the same level as PHOENIX® or API® (data not shown).

*Streptococcus* species are notoriously difficult to be identified and often 16S sequence data are not informative enough to distinguish species. Glazunova et al. [5] have produced phylogenies of this genus inferred from rpoB, sodA, gyrB and groEL sequence comparisons that were more discriminative than those derived from 16S rRNA. This would explain the rather low %AI (71.4%) observed for *Streptococcus* spp. other than *S. agalactiae* and *S. pneumoniae*. MALDI-TOF MS, however, provides reliable identification of species belonging to the *viridans* [6] and *mutans* complex [7]; in the latter case MALDI-TOF MS was useful also for differentiation at the subspecies level. For group A *Streptococcus* (*S. pyogenes*), MALDI-TOF MS was able to distinguish isolates from cases of necrotizing fasciitis from those associated with non-invasive infections, despite they shared the same *emm* type [8]. Additional work is needed for species in this genus, however, for which a careful analysis of regional strains could also be very important. For *Streptococcus* and *Staphylococcus* species we have observed regional differences in isolates characteristics and we are currently establishing a local database for both genera, to define new SuperSpectra that should allow rapid and more reliable identification of local isolates belonging to this variable taxonomic group.

Dubois et al. [9] have already shown that MALDI-TOF MS is a powerful and reliable method to identify *Staphylococcus* species. Most *Staphylococcus* species are correctly identified by MALDI-TOF MS with a high level (>90%) of confidence. According to Bernardo et al. [10] MALDI-TOF MS can be used for rapid identification of clonal strains of *S. aureus*; we are currently investigating the possibility of exploiting this feature to track nosocomial or community acquired methicillin resistant *S. aureus* strains. We cannot exclude that in the near future MALDI-TOF MS may allow discriminating methicillin resistant from sensitive *Staphylococcus* isolates, as suggested by some authors [11], [12], but, probably because of the polyphyletic nature of this resistance [13], we have not yet achieved a robust typing at this level.

This is the first study that has tried to quantify the efficiency of MALDI-TOF MS as compared to currently used methods. We also used a mass spectrometer and a database different from that described in previous publications and we found an almost perfect concordance of our results with those so far reported in the literature. We have been able to show that MALDI-TOF MS represents a rapid, reliable and cost-effective identification technique for clinically relevant bacteria. The method, however, has some limitations. Reliable identification of organisms by this technique is possible only if the spectral database contains data from strains that have been carefully characterised by sequencing specific and informative gene regions (e.g. 16S RNA, gyrB, rpoB, or hsp60 genes for bacteria and ITS regions for fungi). Calibration and selection of internal standards is needed when the work aims at intraspecific characterization. Finally, for a conclusive analysis of region specific strains, careful internal calibration and preparation of locally adapted databases should be considered when geographic variation in the genotypic and phenotypic expression of some taxa (e.g., *Streptococcus* or *Staphylococcus*) is expected.

Overall, MALDI-TOF has found a well-deserved place in bacterial taxonomy. Problems related to the analysis of mixed cultures, presently not yet fully solved, are likely to be overcome in the near future, and attempts to identify pathogens directly in the clinical samples have already yielded promising results [14]. Susceptibility testing of fungi and bacteria by MALDI-TOF may also be soon possible [15]. MALDI-TOF MS may thus become the method of choice for the identification of clinically and environmentally relevant organisms.

## Materials and Methods

### Isolates examined

Bacterial isolates were received during a three-month period from our bacteriological routine laboratory. Isolates were collected in sequential order and no selection criterion was applied to their choice. In a first validation step we analysed 1,019 isolates belonging to 27 bacterial genera (Table 1). Subsequently we studied 202 additional isolates of *Enterococcus*, *Gardnerella* and *Streptococcus* as well as 343 isolates that belonged to 17 *Staphyloccoccus* species.

Isolates were cultured during 24–48 h at 35°C on 5% sheep blood agar, chocolate agar, Mac Conkey agar, and Mueller Hinton agar supplemented with 5% sheep blood (bioMérieux, Lyon, France). Growing colonies were checked for mixed cultures, transferred to a new plate and the isolates growing in pure culture were subsequently conserved in 7% skimmed milk at −80°C until use.

### Identification by biochemical methods and sequencing

#### Biochemical methods

After Gram staining and determination of catalase and oxidase activities, isolates were identified using PHOENIX® identification cards (BD Diagnostics, Sparks, MD, USA) or API® identification strips (bioMérieux, Lyon, France), both according to the manufacturer's instructions. We also used Slidex Staph Plus (bioMérieux, Lyon, France) for *Staphylococcus aureus* identification. Haemolytic streptococci were identified based on the combination of colony morphology, Gram staining and rapid latex agglutination test (Streptex; Remel, Lenexa, KS).

#### Sequence data

Isolates that yielded discrepant results between routine and MALDI-TOF MS identifications were subjected to partial 16S rRNA gene sequencing. Unequivocal identification was defined as the highest sequence homology (99%) with a unique species sequence in GenBank.

DNA extraction was performed with the QIAamp DNA Mini kit (QIAGEN AG, Switzerland) according to the manufacturer's instructions.

Primers used for the amplification of the partial 16S rRNA gene sequence were UNI16SRNA-L (nucleotide sequence 5′-ATTCTTAGAGTTTGATCATGGCTCA-3′) and UNI16SRNA-R (nucleotide sequence 5′-ATGGTACCGTGTGACGGGCGGTGTGTA-3′), which allowed the amplification of a 1400 bp DNA fragment [16], [17].

PCR thermal cycling conditions were 5 min at 95°C for 1 cycle, followed by 35 cycles of 30 sec at 94°C, 30 sec at 52°C, and 1 min at 72°C. The last cycle was performed at 72°C and lasted 10 min. DNA purification was performed using NucleoSpin® (Cat. No. 740609.250) according to the instructions for direct purification of PCR products. We quantified the amplified and purified DNA fragments before the sequencing reaction using a NANO DROP® ND-1000 spectrophotometer (Thermo Fisher Scientific, Houston, USA).

Sequencing reactions were carried out using Big Dye® Terminator v1.1 Cycle Sequencing Kit (Applied Biosystems, Rotkreuz, Switzerland) with a 15 µl total volume composed of 2 µl Big Dye®Terminator, 3 µl Big Dye®buffer, 3 µl primer 1 µM, 6 µl H_2_O and 1 µl DNA sample.

For the sequencing reaction the amplification cycle was 10 sec at 96°C, 5 sec at 50°C, and 4 min at 60°C. Sequence reactions were purified by Sephadex G-25 (Amersham Biosciences, Otelfingen, Switzerland) before sequencing on an ABI 310 Genetic Analyzer (Perkin Elmer Instrument, Applied Biosystems, Rotkreuz, Switzerland).

### MALDI-TOF MS

All samples were analyzed with a MALDI-TOF MS Axima™ Confidence spectrometer (Shimadzu-Biotech Corp., Kyoto, Japan) in positive linear mode (m/z = 2,000–20,000). A small amount of a colony of each pure culture was transferred to a FlexiMass™ target well using a disposable loop, overlaid with 0.5 µl of 2,5-dihydroxybenzoic acid matrix solution (DHB; 10 mg/ml in acetonitrile/0.1% trifluoroacetic acid 1∶1) and air-dehydrated within 1–2 min at 24–27°C.

The reference strain *Escherichia coli* K12 (genotype GM48) was used as a standard for calibration and as reference for quality control. Sample information such as medium and grown conditions was imported into the software Shimadzu Biotech Launchpad™, v.2.8 (Shimadzu-Biotech Corp., Kyoto, Japan). Protein mass profiles were obtained with detection in the linear positive mode at a laser frequency of 50 Hz and within a mass range from 2,000–20,000 Da. Acceleration voltage was 20 kV and extraction delay time 200 ns. A minimum of 20 laser shots per sample was used to generate each ion spectrum. For each bacterial sample, 50 protein mass fingerprints were averaged and processed. Spectra were analyzed using SARAMIS™ (Spectral Archive And Microbial Identification System, AnagnosTec GmbH, Potzdam, Germany), a software in which the identification at the species level is based on a percentage of confidence referred to reference spectra (SuperSpectra™) that contain family, genus and species specific m/z biomarkers, as described in the SARAMIS™ user manual.

### Data analysis

Genetic data were analyzed using the software ABI Prism™ 310 Collection Genetic Analyser (Applied Biosystems, Rotkreuz, Switzerland). Alignments were performed using the BioNumerics software v.6.01 (Applied Maths, Sint-Martens-Latem, Belgium). The modular microorganism identification system AnagnosTec SARAMIS™ was used to archive and evaluate MALDI-TOF MS data. SARAMIS™ was also used to construct dendrograms to show taxonomic relationships among strains.

Agreement of identification (AI) between MALDI and classical methods (API®, PHOENIX®) was defined as the identical outcome of identification of a given isolate by all three methods. Percentage of agreement between identifications (AI%) was computed to compare the agreement of the MALDI-TOF MS identifications with those of the classical methods. 16S rRNA gene sequencing was used as a constructed gold standard only in those cases when a discordant result was observed.

When appropriate, we calculated the estimated sensitivity and specificity and the 95% confidence intervals (CI), as well as the positive predictive (PPV) and negative predictive (NPV) values compared to the constructed perfect standard, corresponding to the identification by 16S gene sequences. We calculated the estimated sensitivity and specificity by defining a positive identification by MALDI-TOF MS when the identification score was at least ≥90%. Sensitivity, specificity and efficiency were computed using DAG_Stat [18].
